# Optimized connectome architecture for sensory-motor integration

**DOI:** 10.1162/NETN_a_00022

**Published:** 2017-12-01

**Authors:** Jacob C. Worrell, Jeffrey Rumschlag, Richard F. Betzel, Olaf Sporns, Bratislav Mišić

**Affiliations:** Department of Psychological and Brain Sciences, Indiana University, Bloomington, Indiana, USA; Department of Cell Biology and Neuroscience, University of California Riverside, Riverside, CA, USA; Department of Bioengineering, University of Pennsylvania, Philadelphia, PA, USA; Montréal Neurological Institute, McGill University, Montréal, Canada

**Keywords:** Connectome, Brain, Spreading, *Drosophila*

## Abstract

The intricate connectivity patterns of neural circuits support a wide repertoire of communication processes and functional interactions. Here we systematically investigate how neural signaling is constrained by anatomical connectivity in the mesoscale *Drosophila* (fruit fly) brain network. We use a spreading model that describes how local perturbations, such as external stimuli, trigger global signaling cascades that spread through the network. Through a series of simple biological scenarios we demonstrate that anatomical embedding potentiates sensory-motor integration. We find that signal spreading is faster from nodes associated with sensory transduction (sensors) to nodes associated with motor output (effectors). Signal propagation was accelerated if sensor nodes were activated simultaneously, suggesting a topologically mediated synergy among sensors. In addition, the organization of the network increases the likelihood of convergence of multiple cascades towards effector nodes, thereby facilitating integration prior to motor output. Moreover, effector nodes tend to coactivate more frequently than other pairs of nodes, suggesting an anatomically enhanced coordination of motor output. Altogether, our results show that the organization of the mesoscale *Drosophila* connectome imparts privileged, behaviorally relevant communication patterns among sensors and effectors, shaping their capacity to collectively integrate information.

## Author Summary

The complex network spanned by neurons and their axonal projections promotes a diverse set of functions. In the present report, we study how the topological organization of the fruit fly brain supports sensory-motor integration. Using a simple communication model, we demonstrate that the topology of this network allows efficient coordination among sensory and motor neurons. Our results suggest that brain network organization may profoundly shape the functional repertoire of this simple organism.

## INTRODUCTION

Recent advances in imaging of neural circuits have resulted in detailed maps of neural elements and their connections (Lichtman & Denk, 2011). Topological analysis of these “connectomes” has revealed several organizational features that appear to be conserved across spatial scales (Betzel & Bassett, 2016; Sporns, 2014) and phylogeny (van den Heuvel, Bullmore, & Sporns, 2016), including the existence of functionally specialized modules (Rubinov, Ypma, Watson, & Bullmore, 2015; Shih et al., 2015; Varshney, Chen, Paniagua, Hall, & Chklovskii, 2010) bound together by an integrative core of highly connected hub nodes (Bota, Sporns, & Swanson, 2015; Towlson, Vértes, Ahnert, Schafer, & Bullmore, 2013; van den Heuvel, Kahn, Goñi, & Sporns, 2012; Zamora-López, Zhou, & Kurths, 2010). A significant remaining challenge is to understand how the organization of neural circuits supports emergent functional interactions and adaptive behavior (Mišić & Sporns, 2016; Vogelsteinet al., 2014).

Theoretical models that describe the unfolding of communication processes through anatomical pathways hold great promise to bridge the gap between static anatomical features and global dynamical interactions. A spectrum of frameworks and models, em phasizing communication via shortest paths (van den Heuvel et al., 2012), ensembles of paths and walks (Avena-Koenigsberger et al., 2017; Crofts & Higham, 2009), diffu sion (Abdelnour, Voss, & Raj, 2014; Bacik, Schaub, Beguerisse-Díaz, Billeh, & Barahona, 2016; Mišić, Goñi, Betzel, Sporns, & McIntosh, 2014; Mišić, Sporns, & McIntosh, 2014), and sustained coherent oscillations (Deco, Jirsa, McIntosh, Sporns, & Kötter, 2009; Gollo, Zalesky, Hutchison, van den Heuvel, & Breakspear, 2015) have begun to link anatomical connectivity patterns with emergent activity patterns. For instance, diffusion models have been used to predict statistical associations (functional connectivity) between distributed areas (Abdelnour et al., 2014; Goñi et al., 2013), as well as the clustering of functional brain networks into coherent modules (Atasoy, Donnelly, & Pearson, 2016; R. Betzel et al., 2013). Altogether, these studies suggest that integrative properties of brain networks and specific brain regions naturally arise from their anatomical connectivity and topological embedding (Bacik et al., 2016).

In the present report we investigate the spreading and subsequent integration of neural signals in the mesoscale *Drosophila* connectome, previously reconstructed from 12,995 images of individual neurons (Shih et al., 2015). Neurons are grouped into 49 distinct populations, termed local processing units (LPUs), that serve as the nodes of the network. We apply a simple model of collective spreading that describes how local perturbations trigger global activity cascades that propagate across the network (linear threshold model) (Granovetter, 1978; Mišić et al., 2015; O’Dea, Crofts, & Kaiser, 2013; Watts, 2002). We consider three biological scenarios: (a) how a single perturbation develops into a single cascade, (b) how two signals synergistically spread through the network, and (c) how two signals, endowed with different content, spread and ultimately integrate with one another. To investigate whether anatomical embedding potentiates integration, we focused on several test cases directly related to sensory-motor integration. We tested the hypothesis that signals initiated in neuronal populations associated with sensory transduction (sensors) would spread to populations associated with motor output (effectors) more effectively than to other targets. Furthermore, we hypothesized that, in order to produce coordinated action patterns, the architecture of the *Drosophila* connectome would potentiate the convergence of signals towards effector nodes.

## RESULTS

### Modules

**Figure 1.**
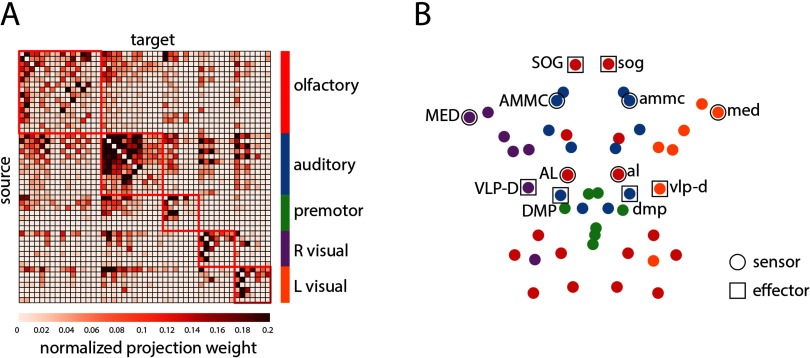
Modular organization of the *Drosophila* connectome. (A) *Drosophila* connectome adjacency matrix derived from the FlyCircuit 1.1 database. Modules were estimated using multiresolution community detection. The nodes of the adjacency matrix are ordered by community assignment. (B) Axial view of the network’s spatial layout. LPUs involved in sensory signal transduction (sensors) and motor execution (effectors) are outlined with circular and square markers, respectively.

Multiscale community detection revealed five modules: olfactory, auditory or mechano sensory, premotor, right visual, left visual (Figure 1). The recovered communities were identical to the communities reported by Shih et al. (2015), save for the assignment of the left and right optic tubercle (optu/OPTU), which were placed in the central auditory/mechanoreceptive module, rather than the left and right visual modules. To facilitate comparisons with previous reports, the optu and OPTU LPUs were manually reassigned to the left and right visual modules, yielding the same partition as first reported in Shih et al. (2015).

### Sensor-Effector Spreading

**Figure 2.**
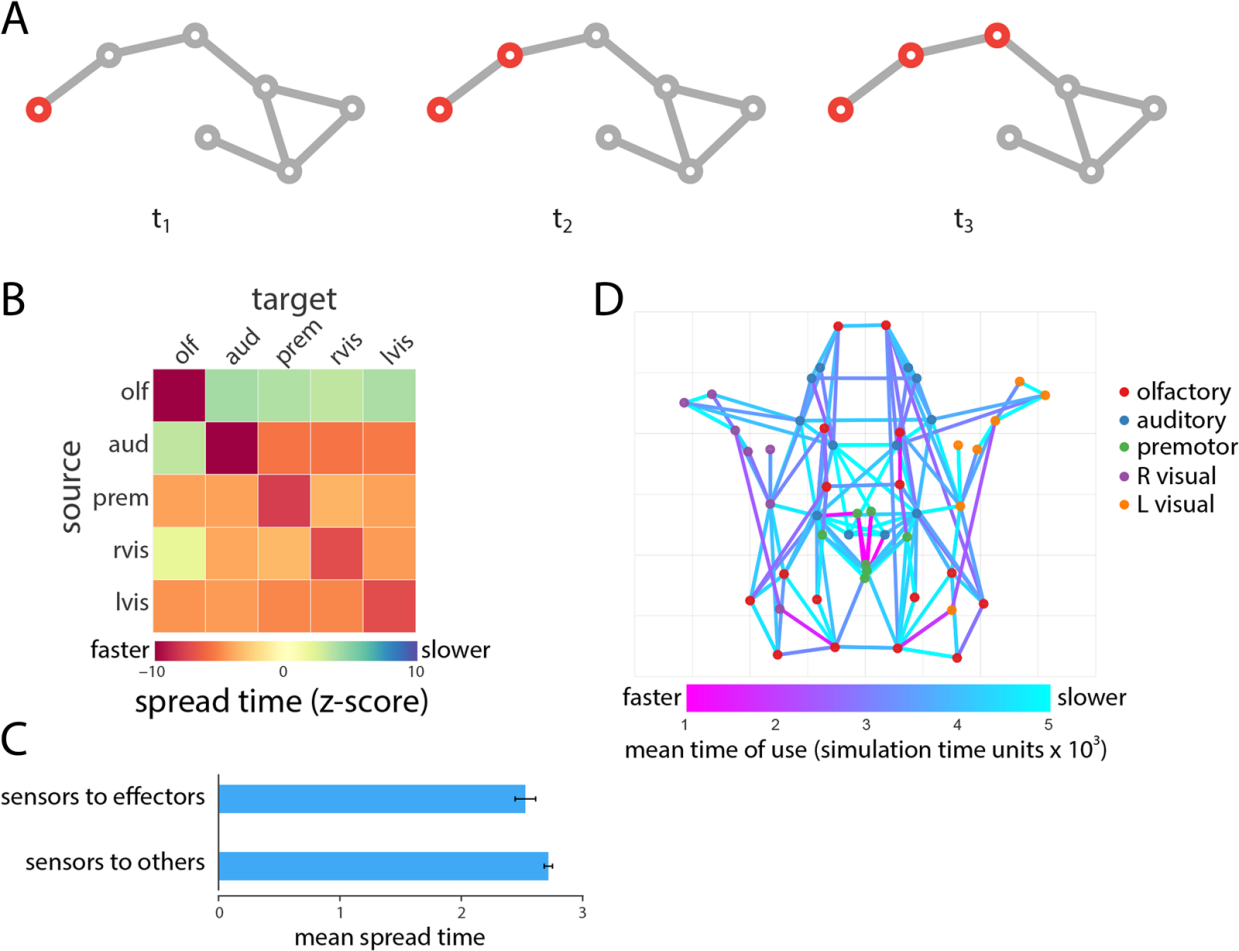
Single cascade spreading. (A) Schematic showing the spread of a single cascade. (B) Mean spread time for all seed-target combinations, stratified by module membership. (C) Test case comparing spread times from sensor to effector nodes versus sensor to noneffector nodes. (D) Projections most commonly used to spread cascades and the average time at which they are used.

We initially consider the scenario where a single perturbation triggers a global cascade that spreads through the network (Figure 2A). The perturbation may represent the transduction of an external stimulus or some endogenous event, such as synchronized postsynaptic potentials in a neuronal ensemble. The spread time is the time (in dimensionless units) that it takes for a signal initiated at some source node to reach a target node.

We first seek to build intuition about how spread times and the overall performance of the spreading model relate to more traditional network measures. Seed node out-strength was significantly correlated with mean spread time to the rest of the network (*r* = −0.48, *p* = 5.6 × 10^−4^), suggesting that perturbations initiated at nodes with stronger outputs spread faster. Similarly, target node in-strength was significantly correlated with mean spread times across all seed nodes (*r* = −0.78, *p* = 3.5 × 10^−11^), indicating that target nodes with more inputs adopted the active state faster. Finally, we find that the communicability between seed and target nodes, corresponding to a weighted sum of all walks between them (Crofts & Higham, 2009; Estrada & Hatano, 2008), was also a significant predictor of spread time between those nodes (*r* = −0.42, *p* ≈ 0). In other words, source-target pairs that have greater communicability (indicating that they are, on average, connected by shorter paths and walks) also have faster spread times.

We next investigate spreading within and between modules (Figure 2B). For each source and target module, we first calculate the mean spread time among all constituent nodes. We then express this quantity as a z-score relative to a null distribution obtained by randomly permuting module labels and recalculating mean spread times. We find that spread times are generally faster in the empirical network (corresponding to negative z-scores), suggesting that the modular organization of the *Drosophila* connectome may be optimized for rapid communication. Fastest spreading was observed within modules, consistent with the definition of modules as communities of nodes with high mutual connection density (Nematzadeh, Ferrara, Flammini, & Ahn, 2014).

To address the hypothesis that the organization of the *Drosophila* connectome should potentiate information transmission from sensors to effectors, we compared the mean spread time from sensor nodes to effector nodes with spread time from sensor nodes to noneffector nodes. Consistent with the hypothesis, we find that spreading from sensors to effectors is significantly faster compared with spreading from sensors to noneffectors (Wilcoxon *p* = 0.0011; Cohen’s *d* = 0.35; Figure 2C).

Finally, we investigate the contribution of individual projections to global spreading patterns (Figure 2D). We define the transit time associated with each projection as the ratio of the Euclidean distance spanned by that projection and the weight of the projection. We then use asynchronous updating to infer the contributions of specific projections to the activation of specific nodes (see Methods section). Figure 2D shows the most commonly used projections across all *n* = 49 possible seeding scenarios, as well as the mean time (in dimensionless simulation time units) at which those projections were used. The projections and subnetworks they delineate bear a close correspondence to the putative rich club of the *Drosophila* connectome (bilateral DMP, VMP, VLP-D, SDFP, and FB in the central brain), encompassing many of the strongest projections and integrative LPUs in the network (Shih et al., 2015). Statistically, connections between rich club nodes were used earlier and more often compared with other connections (*p* = 0.05 and *p* ≈ 0 for both measures, Cohen’s *d* = 0.43 and 0.97, respectively), suggesting that this central collective of high-strength nodes is disproportionately more involved in signal spreading.

### Synergistic Relationships Among Sensors

**Figure 3.**
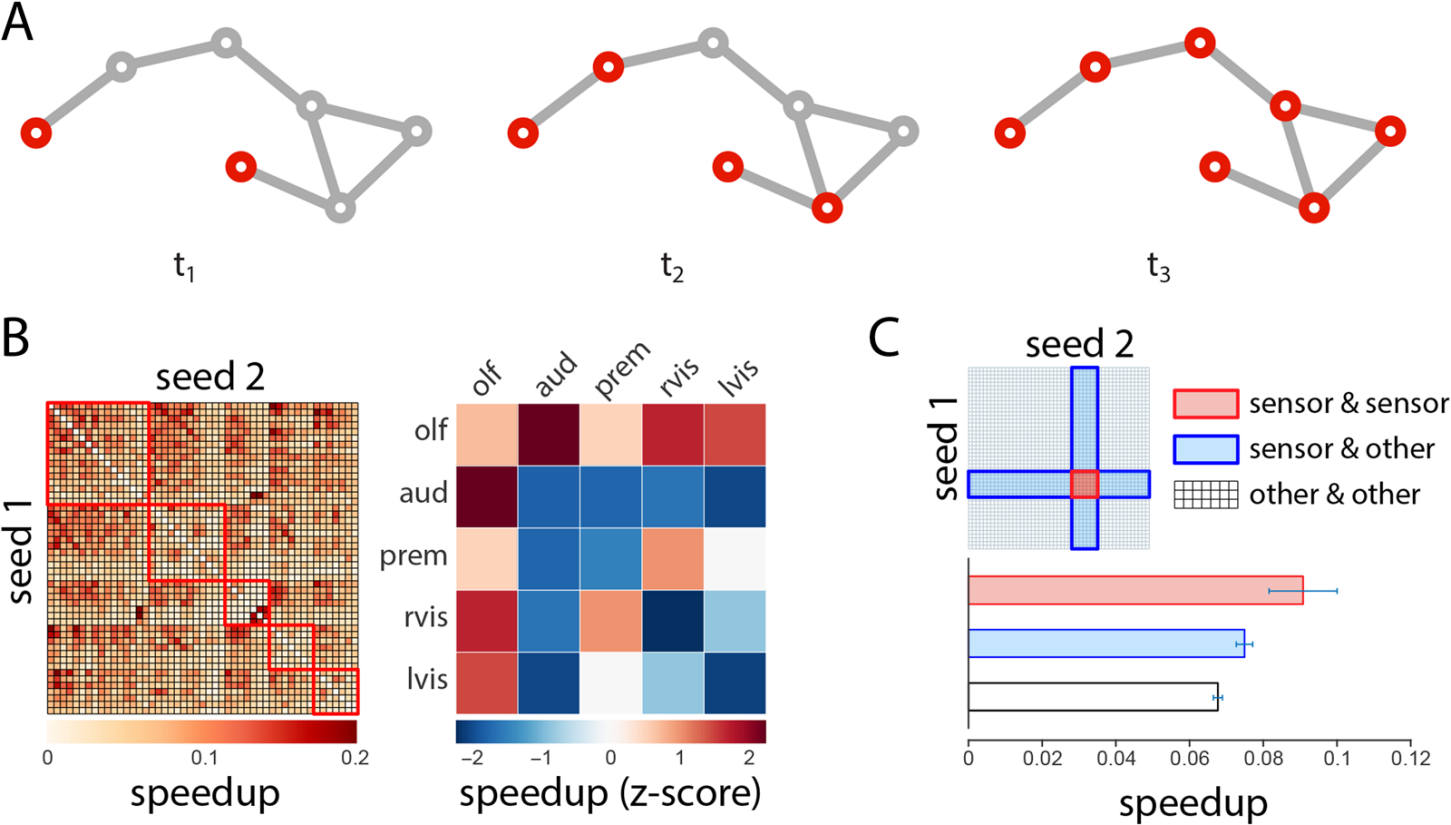
Synergistic spreading. (A) Schematic showing the spread a signaling cascade initiated in two seed nodes. (B) The percentage speedup in spread time for the whole network, shown for all possible two-seed combinations. (C) Test cases comparing speedups for cascades initiated at two sensor seed nodes, versus cascades initiated at one sensor and one nonsensor node, and two nonsensor nodes.

We next investigate the effects of initiating a cascade simultaneously in two seed nodes (Figure 3A). In certain instances, introducing a perturbation at two seed nodes may theoret ically accelerate the spread of the cascade across the network. We operationalize the synergistic benefit of simultaneous perturbations as the percentage speedup in spread time for the two-seed scenario compared with the faster of the two individual one-seed scenarios (see Methods).

The speedups for every two-seed combination are shown in matrix form in Figure 3B (left). The results suggest that simultaneous perturbations are less effective when triggered in the same module and more effective when triggered in different modules (Wilcoxon *p* = 6.69 × 10^−5^), consistent with the definition of modules as densely interconnected communities. We then calculate the mean within- and between-module speedups and express them as z-scores relative to a null distribution that was constructed by randomly permuting module assignments for individual nodes (Figure 3B right). Comparison with the null model suggests several greater than expected cooperative effects, including between the higher-order olfactory and auditory modules (permuted *p* = 0.016), indicating an enhanced potential for functional coupling among these two modules.

Figure 3C shows a set of test cases in which we focus on the cooperative effects among pairs of sensor LPUs. We hypothesize that, if the architecture of the network has evolved to support multisensory integration, sensor LPUs may show greater synergy with each other than with other nodes in the network. We thus compare speedups for two-sensor pairs (sensor and sensor) with speedups for sensor and nonsensor nodes (sensor and other), as well as nonsensor pairs (other and other). The speedup for sensor pairs is greater than for sensor/nonsensor pairs (Wilcoxon *p* = 0.051, Cohen’s *d* = 0.44) and for nonsensor pairs (*p* = 0.007, *d* = 0.62), but is statistically significant only for the latter comparison.

### Signal Convergence at Effectors

**Figure 4.**
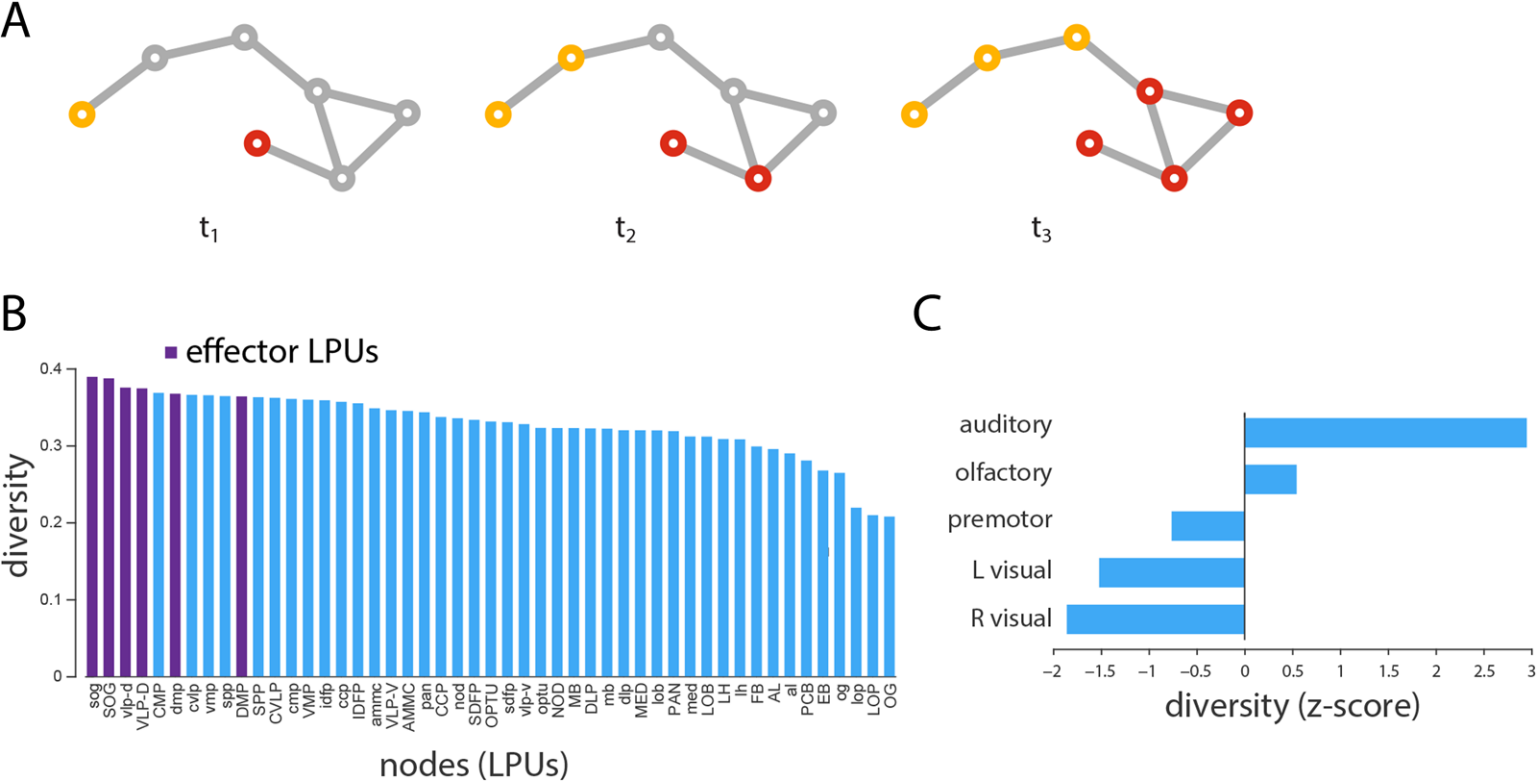
Signal convergence. (A) Schematic showing the propagation and convergence of two signaling cascades. (B) Mean neighborhood diversity of all nodes, calculated across all possible two-seed scenarios. (C) Mean neighborhood diversity for each module, expressed as a z-score relative to a label-permuting null model.

We next investigate the scenario in which two perturbations, carrying different signals, develop into competing cascades (Figure 4A). This scenario allows us to characterize how separate signaling cascades develop and to determine where they converge. To estimate the likely convergence points between cascades, we define the diversity of individual nodes’ neighborhoods as the entropy of their neighborhood vector (see Methods). Nodes whose neighbors adopt the same state and are part of the same cascade will have low diversity, while nodes whose neighbors are distributed among the two cascades will have high diversity.

Across all possible seeding scenarios, we find in-strength to be significantly correlated with diversity (*r* = 0.50, *p* = 2.8 × 10^−4^), suggesting that highly connected nodes are better positioned to mediate the integration of multiple signals. Interestingly, the six effector LPUs (sog, SOG, vlp-d, VLP-D, dmp, and DMP) ranked as the top first, second, third, fourth, sixth, and tenth most diverse nodes (Figure 4B), suggesting that the *Drosophila* connectome is organized to maximize the convergence and, presumably, integration of information prior to motor output. At the modular level, we find that modules contributing significantly to the highly central rich club, such as the auditory and olfactory modules, have the greatest average diversity (Figure 4C).

### Coactivation of Effectors

**Figure 5.**
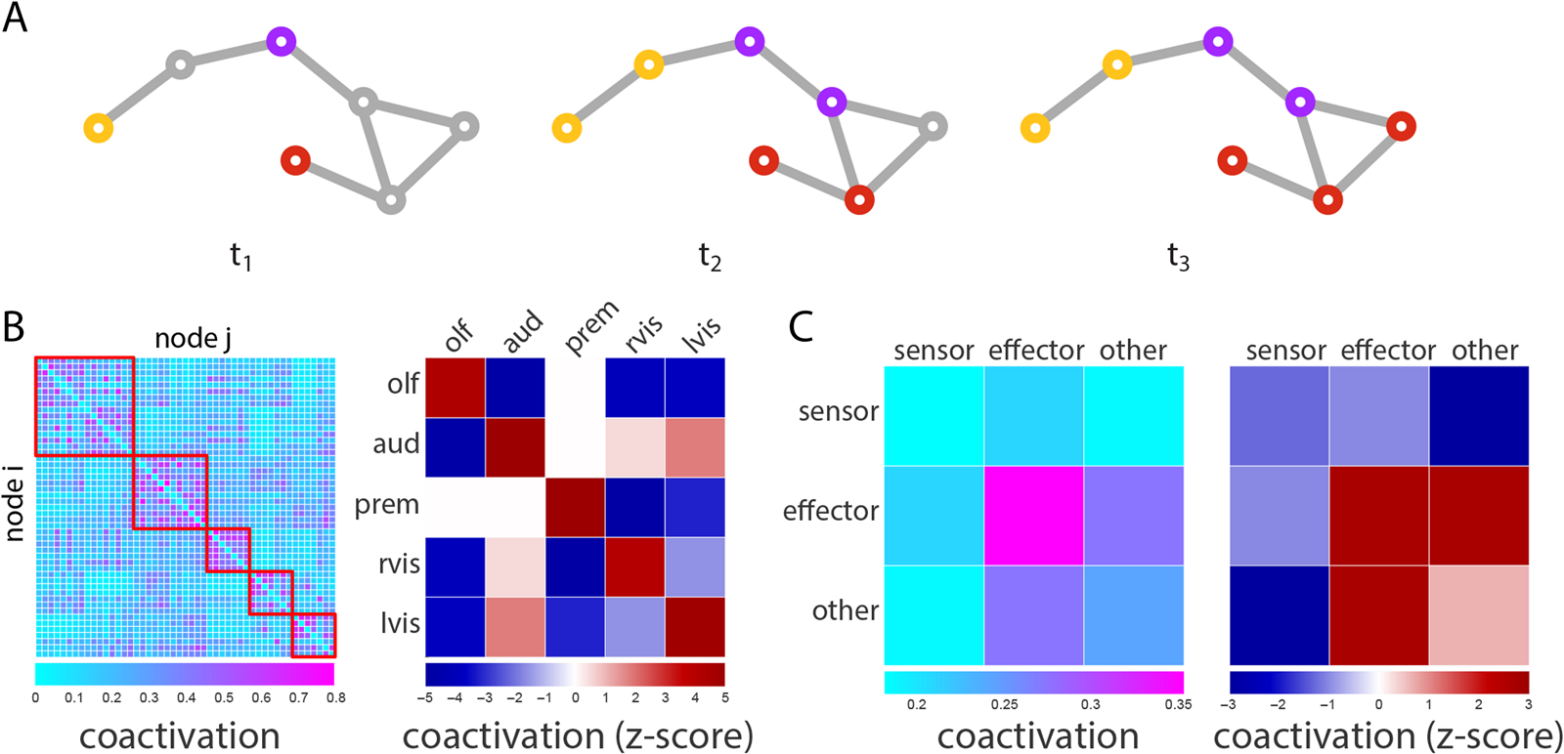
Co-activation patterns. (A) Schematic showing the spread of three competing cascades. (B) Propensity for two nodes to adopt the same signal (coactivation). Mean coactivations within and between modules are expressed as z-scores relative a label-permuting null model. (C) Mean coactivation for all combinations of sensor, effector and nonsensor, noneffector (other) nodes. Mean coactivations are also expressed as z-scores relative to a label-permuting null model.

Finally, we investigate the scenario in which more than two perturbations are introduced, each carrying a different signal (e.g., an auditory and a visual stimulus; Figure 5A). To estimate the propensity for two nodes to coactivate, we calculate the proportion of time a given node pair adopts the same signal across all possible seeding scenarios. Node pairs that coactivate often are presumably more likely to engage in common or overlapping functions. Indeed, pairs of nodes belonging to the same module tend to display greater coactivation than pairs belonging to different modules (Figure 5B). Post hoc comparison confirmed that coactivation of nodes within the same module was greater than coactivation of nodes in different modules (Wilcoxon *p* = 9.37 × 10^−50^).

Stratifying nodes into sensors and effectors, we find that the greatest coactivation is observed for pairs of effectors, compared with pairs of sensors, pairs of sensors and effectors, and pairs of sensors and other (nonsensor and noneffector) nodes (*p* ≈ 0, *p* = 0.001, *p* ≈ 0;*d* = 1.21,1.42,1.38, respectively; Figure 5C), suggesting the wiring of the *Drosophila* connectome potentiates coordination among effectors by allowing common inputs to converge to out put LPUs. Comparison with a label-permuting null model further suggests that this enhanced effector-effector coactivation is driven by topological organization (permuted *p* = 0.0013; Figure 5C). Interestingly, coactivation among other classes of nodes, such as pairs of sensors, is lower than expected according to the null model. This suggests that the wiring of the *Drosophila* connectome may also minimize some types of functional interactions, effectively slowing integration or mixing of signals in areas where segregation of function may be more important, such as sensor nodes.

## DISCUSSION

The present study provides further evidence that the anatomical connectivity of brain networks supports specific modes of neural signaling, giving rise to highly organized spreading patterns. Using a simple spreading model, we demonstrate that (a) topology potentiates spreading from sensors to effectors; (b) topology potentiates synergistic relationships among sensors; and (c) information flow converges towards effectors, facilitating integration and coordination prior to motor output. The scenarios presented here, while implemented in a formally simple model of spreading processes in networks, offer a useful framework for testing specific hypotheses about interactions in neural circuits.

While the propensity for anatomical pathways to shape functional interactions is well doc umented both in computational models (Deco et al., 2009; Goñi et al., 2013; Honey et al., 2009) and in empirical data (Honey et al., 2009; Mišić et al., 2016; Shen et al., 2015), a key question is how topological characteristics shape signal propagation. Our results suggest that signal spreading is strongly influenced by node strength and by the community structure of the network. Across all spreading scenarios, highly connected nodes and their mutual projections were disproportionately more involved in signal spreading. This is consistent with the notion that “hub” nodes promote information transfer and synchronization among remote neuronal populations, enabling a diverse repertoire of cognitive functions (Bertolero, Yeo, & D’Esposito, 2015; Crossley et al., 2013; Gollo et al., 2015; van den Heuvel & Sporns, 2013; Váša et al., 2015).

Likewise, the modular specialization of the network, reflected in its community structure, imparts a distinct signature on the observed spreading patterns. Two of the central, higher modules (olfactory and auditory) exhibited a tendency for synergistic interactions, suggesting a topological predisposition for cooperative function. In addition, LPUs in these modules were more likely to occupy positions at the border or intersection of multiple cascades, suggesting that their anatomical embedding naturally predisposes them to integrate information from multiple sources.

More generally, these results highlight how organizational features persist across spatial scales (Betzel & Bassett, 2016; Lichtman & Denk, 2011). Clear roles have been observed for rich clubs/cores and for modules, both at the microscopic (Shih et al., 2015; Towlsonet al., 2013; Varshney et al., 2010) and macroscopic scales (Bota et al., 2015; Hagmann et al., 2008; Harriger, van den Heuvel, & Sporns, 2012; Rubinov et al., 2015; van den Heuvel et al., 2012; Zamora-López et al., 2010. Our results complement these past studies by showcasing the possible dynamical importance of these architectural features.

The importance of anatomical embedding is salient in sensor-effector relationships. Signal cascades originating from sensor nodes propagated more rapidly to effectors than to other nodes, suggesting that communication between these classes of nodes may be privileged, ensuring a rapid transition from perception to action. Furthermore, signal propagation was supra-additive (i.e., accelerated) when signals were introduced at pairs of sensors, suggesting that simultaneous activation of sensors would result in accelerated signaling through the network.

Further downstream, effectors appeared to be the main points of convergence for multiple cascades. This anatomically driven funneling of information towards motor LPUs suggests that the *Drosophila* connectome may be organized to maximize integration prior to motor output, possibly to allow for sustained control and adjustment. In addition, pairs of effectors displayed an enhanced potential to coactivate, perhaps reflecting an anatomical organizational principle to facilitate motor coordination. Interestingly, the topological importance of motor neurons has also been reported in the nematode *C. elegans*, where synthetic ablations of motor neurons are associated with the greatest functional disruptions (Bacik et al., 2016). Altogether, these results demonstrate how simple tractable models can be used to test the effects of focal perturbations applied to specific regions of interest.

A notable methodological limitation is the choice of threshold parameter. As there is no external metric by which to evaluate the threshold (e.g., correspondence with functional connectivity), we employed a heuristic. Namely, we chose the largest possible threshold at which all perturbations elicit complete cascades, in order to facilitate comparisons among all nodes. In the future, we foresee three alternative criteria by which to select the threshold. First, thresholds could be selected with respect to a measure of model fit, such as the correspondence between spreading patterns and empirical functional connectivity patterns. Second, thresholds could be selected to favor certain types of dynamics. As we discuss in the Methods section, at greater thresholds cascades spread via walks outside of shortest paths, akin to a diffusion process. Third, investigators could allow for the possibility that some cascades do not spread through the whole network, and instead use the size of the cascade as the dependent variable in the model, rather than spread velocity. This is commonly done with the linear threshold model (LTM) (Nematzadeh et al., 2014) and would permit exploration of a wider range of thresholds.

The present model can offer novel insight into the design principles of neural circuits, but it does so by sacrificing potentially significant physiological detail. This approach is similar to other models of naturally occurring complex systems with collective dynamics, such as flocking and swarming behavior in animals (Couzin, Krause, Franks, & Levin, 2005; Vicsek, Czirók, Ben-Jacob, Cohen, & Shochet, 1995), metropolitan traffic patterns (Helbing, Farkas, & Vicsek, 2000), disease epidemics (Pastor-Satorras & Vespignani, 2001), and human social interactions (Schelling, 1971), all of which emphasize the emergent properties of systems under study at the expense of microscopic detail. This type of approach has recently been adopted in systems neuroscience as well, where simple models have been used to gain insight into the emergence of functional interactions (Deco, Senden, & Jirsa, 2012; Fraiman, Balenzuela, Foss, & Chialvo, 2009; Stramaglia et al., 2017).

In particular, it is worth considering what specific neurobiological phenomena this model can and cannot represent. The modeled spreading patterns may be thought of as episodes of synchronized, coherent communication (Beggs & Plenz, 2003; Fries, Reynolds, Rorie, & Desimone, 2001; Womelsdorf et al., 2007; Zhou, Zemanová, Zamora, Hilgetag, & Kurths, 2006). For instance, if several afferent projections to a neuronal population emanate from populations that are themselves mutually synchronized, they will act as an external synchronizing force on that population. Thus, the model captures how neuronal populations may generically influence each other via direct and indirect projections. Compared with other types of neural models (Deco, Jirsa, Robinson, Breakspear, & Friston, 2008), the present spreading model does not explicitly embody physiological parameters, such as firing rates or membrane conductance, but serves as a tool to characterize the architecture and dynamic potential of neural circuits.

Another significant limitation is that the LTM models only the initial spreading pattern originating from a perturbation, and cannot be used to model any subsequent feedback or time-dependent reconfiguration of functional interactions. In this sense, the current model may be thought of as a single, transient episode of synchrony following a perturbation. A simple yet significant addition to the model would be to include a refractory state, mimicking the refractory period of single neurons. In that case, the model becomes the well-studied Susceptible-Infected-Recovered-Susceptible (SIRS) model, a family of epidemiological models that have also been successfully applied in the study of brain activity (Gollo, Copelli, & Roberts, 2016; Haimovici, Tagliazucchi, Balenzuela, & Chialvo, 2013). While the addition of refractory periods introduces rich time-dependent dynamics and broadly conforms to single neuron biophysics, it remains to be determined whether such models and the refractory periods they capture are applicable to populations of neurons and neuronal ensembles.

More generally, it is worth noting that the LTM belongs to a broader class of contagion models, whereby the state of a node depends on the state of its neighbors. These models range from binary decisions in networked systems, such as the diffusion of influence or technological innovations (Valente, 1995), to models of epidemic spreading (Anderson, May, & Anderson, 1992), bootstrap percolation (Adler, 1991), and self-organized criticality (Bak, Tang, & Wiesenfeld, 1987). Unlike bootstrap percolation and self-organized criticality, where activation depends on the absolute number of activated neighbors, in the LTM activation depends on the fraction of activated neighbors. In the most general case, the LTM can be conceptualized from the perspective of standard percolation (Watts, 2002). Percolation is a convenient model of interactions on complex topologies and refers to the probability of the existence of a path between all nodes in a graph (Saberi, 2015). The relationship between the LTM and percolation is therefore straightforward: The susceptibility of the network to global cascades following a focal perturbation simply depends on the existence of a percolating vulnerable cluster, that is, a subgraph of nodes with degrees less than or equal to the inverse of the threshold parameter (Watts, 2002).

In summary, the present report offers a framework for studying communication processes in neural circuits. As advances in imaging and tract tracing techniques propel connectomics towards comprehensive maps of neuronal connectivity across multiple scales and for multiple organisms, there is a need for general theoretical models that describe the evolution of communication processes on the anatomical substrate. Our results add to a growing literature that the organization of neural circuits may be optimized for specific functions, including sensory-motor integration.

## METHODS

### *Drosophila* Connectome

The *Drosophila* connectome was reconstructed from the FlyCircuit 1.1 database (Chiang et al., 2011; Shih et al., 2015), utilizing images of 12,995 projection neurons in the female *Drosophila* brain. Single neurons were labeled with green fluorescent protein (GFP) using genetic mosaic analysis with a repressible cell marker. GFP-labeled neurons were then delineated from whole brain three-dimensional images. Individual GFP-labeled neurons from each image were coregistered to a female template brain using a rigid linear transform. Individual neurons were stratified into 49 local populations with distinct morphological and functional characteristics, termed local processing units (LPUs), which constituted the nodes of the network. Specifically, LPUs were delineated as neuronal populations with their own population of local interneurons, whose fibers are limited to that LPU (Shih et al., 2015). The resulting connectome is represented as a weighted, directed adjacency matrix.

### Multiscale Community Detection

The modular structure of the network was estimated using the Louvain algorithm (Blondel, Guillaume, Lambiotte, & Lefebvre, 2008), as implemented in the Brain Connectivity Toolbox (Rubinov & Sporns, 2010). Briefly, the goal of the analysis was to identify communities of LPUs that are more densely interconnected with each other than expected. This constraint was operationalized in terms of the modularity *Q* (Leicht & Newman, 2008; Newman & Girvan, 2004):

$$Q(\gamma )=\underset{ij}{\sum }[w_{ij}-\gamma \cdot \rho_{ij}]\delta (\sigma_{i},\sigma_{j}),$$ (1)

where *w*_*ij*_ is observed connection weight between nodes *i* and *j*, while *ρ*_*ij*_ is the expected connection weight between those nodes. In the present study, the expected connection weight between pairs of nodes was defined according to a configuration model, in which node strengths are preserved exactly but where connections are otherwise formed at random, giving

$$\rho_{ij}^{\pm }=\frac{s_{i}^{in}s_{j}^{out}}{2m}.$$ (2)

Here, $s_{i}^{in}$ and $s_{j}^{out}$ are the in- and out-strengths of nodes *i* and *j*, and 2*m* represents the total density of the network. Variables *c*_*i*_ and *c*_*j*_ are the community assignments of nodes *i* and *j*. The Kronecker delta function, (*c*_*i*_, *c*_*j*_), is equal to 1 when the arguments *c*_*i*_ and *c*_*j*_ are equal, and 0 otherwise, ensuring that modularity is only computed for pairs of nodes belonging to the same community. The resolution parameter *γ* scales the relative importance of the null model *ρ*_*ij*_, potentiating the discovery of larger (*γ* < 1) or smaller communities (*γ* > 1).

We scanned the resolution parameters *γ* = 0.5 to *γ* = 2, in increments of 0.05. At each scale, the Louvain algorithm was run 250 times to find a partition that maximized the modularity function (Blondel et al., 2008). To select an appropriate scale, we compared the mean modularity of partitions derived for the empirical network with the mean modularity of partitions derived for a population of 1,000 randomized networks, with the tacit assumption that the optimal scale is one where the average *Q* of the empirical network deviates from the average *Q* of randomized networks to the greatest extent (Bassett et al., 2013). The randomized networks were created using a link-swapping algorithm that preserved the in-degree, out-degree, and out-strength sequences of the network. The greatest difference was observed at resolution *γ* = 1.05, yielding five communities or modules.

### Rich Club Detection

A consistent finding in connectomics across a range of species is the tendency for high-degree nodes to be densely interconnected with each other, beyond what would be expected on the basis of their degrees alone (van den Heuvel et al., 2016). This tendency for nodes “rich” in connectivity to preferentially connect with each other leads to them being referred to as a “rich club” (Colizza, Flammini, Serrano, & Vespignani, 2006). This architectural feature has been posited as a critical component for integrating and disseminating signal traffic (Mišić, Sporns, et al., 2014; van den Heuvel et al., 2012) and stabilizing interareal functional interactions (Gollo et al., 2015; Mišić et al., 2016).

In the present study we use the rich club stratification of Shih et al. (2015), which was derived from the same network. Nodes that were classified as being part of the putative rich club were bilateral DMP, VMP, VLP-D, and SDFP, as well as FB in the central brain. Briefly, rich club detection was performed over a range of degrees *k*. Nodes with degree > *k* are selected and the rich club coefficient, *ϕ*(*k*), is calculated as the density of the resulting subgraph. This procedure is then repeated for a population of randomized networks with preserved density and in- and out-degree sequences (Maslov & Sneppen, 2002), generating a null distribution of rich club coefficients *ϕ*(*k*)_*random*_ at a particular level *k*. This null distribution is then used to normalize the coefficient derived for the original network, yielding a normalized rich club coefficient *ϕ*(*k*)_*norm*_ = *ϕ*(*k*)/*ϕ*(*k*)_*random*_. A rich club is then defined as a set of nodes with degree ≥ *k* over which *ϕ*(*k*)_*norm*_ is consistently greater than 1.

### Communicability

Communicability (*C*_*ij*_) between two nodes *i* and *j* is a weighted sum of all paths and walks between those nodes (Estrada & Hatano, 2008). For a binary adjacency matrix **A**, communicability is defined as

$$C_{ij}=\overset{\infty }{\underset{n=0}{\sum }}\frac{{\left[{\text{A}}^{n}\right]}_{ij}}{n!}={\left[e^{\text{A}}\right]}_{ij},$$ (3)

with walks of length *n* normalized by *n*!, such that shorter, more direct walks contribute more than longer walks. This concept can be generalized to weighted networks, but requires normalization to mitigate the influence of high-strength nodes (Crofts & Higham, 2009). Following Crofts & Higham (2009), this was accomplished by defining a “reduced” adjacency matrix

$${\text{A}}_{red}={\text{S}}^{-1/2}\text{W}S^{-1/2},$$ (4)

where **W** is the weighted adjacency matrix and **S** is a diagonal matrix of node strengths.

### Linear Threshold Model

The linear threshold model describes how a perturbation introduced at one or more seed nodes develops into a cascade and spreads through a network (Granovetter, 1978; Watts, 2002). The perturbation and subsequent cascade are modeled as an active state; any given node adopts this active state only if a certain threshold proportion of its neighbors have also adopted the active state. A family of simple models of collective behavior, LTMs have been extensively studied over a wide range of networks, including spatially embdedded brain networks (Kaiser, Goerner, & Hilgetag, 2007; Kaiser & Hilgetag, 2010; Mišić et al., 2015; O’Dea et al., 2013). The models capture how generic focal perturbations, such as the transduction of a sensory stimulus, spread through connected neuronal populations (see the Discussion section for a discussion of the neurobiological interpretation and limitations).

Formally, the state of a node *i* at time *t* is denoted as a binary variable $r_{i}(t)=\left\{0,1\right\}$, with only two possible states: active (1) or inactive (0). At initialization (*t* = 0), the entire network is inactive, except for a subset of activated seed nodes. The model is then updated synchronously at each time step according to the following rule:

$$r_{i}(t+1)=\left\{\begin{array}{ll} 1 & \text{if}\theta s_{i}<\sum_{j\in N_{i}}r_{j}(t)\\ 0 & \text{otherwise}. \end{array}\right.$$ (5)

Thus, at each time step the state of node *i* depends on its neighborhood, $N_{i}$, and specifically on the number of incident connections (in-degree or in-strength, *s*_*i*_). The node adopts the active state only if the proportion of inputs from active nodes exceeds the threshold *θ*. In the case of binary networks, the threshold represents the proportion of a node’s neighbors that must be active to propagate the cascade. The model can be naturally extended to weighted networks, whereby the threshold represents the proportion of a node’s total weighted inputs (in-strength) that must be connected to active neighbors. In all scenarios, the fundamental performance measure is the adoption or spread time $A_{i\to k}$, from seed node *i* to target node *k*.

The threshold parameter was chosen to satisfy two criteria. At lower thresholds, nodes require fewer neighbors to be active at time *t* in order to become active themselves at time *t* + 1. Thus, nodes will be activated at the earliest possible time step, and the cascade will effectively propagate along the shortest path. As the threshold is increased beyond the inverse of the highest degree/strength in the network, cascades can no longer influence the most highly connected nodes and do not spread through the whole network. Because we sought to compare spreading times for all possible seed-target combinations, we set the threshold to the highest value at which all perturbations will cause a complete cascade (*θ* = 0.01). As we discuss in more detail in the Results section, at this threshold cascades will spread to all target nodes, but their trajectory is only partially predicted by shortest path length (*r* = 0.34, *p* ≈ 0), with much of the spreading process occurring via alternative paths as well.

Note that the activation of a target node depends on three factors: target node in-strength, source node out-strength, and topology. The relative importance of these factors depends on the threshold. Specifically, at higher thresholds it is more difficult to activate nodes, as more of their neighbors need to be active, so the dynamics are more dependent on local connectivity. At lower thresholds, the dynamics are less constrained by local connectivity and more influenced by global topology. Thus, by limiting the threshold to allow spreading to all target nodes, the dynamics are also more likely to be shaped by global topology.

The order in which individual projections contribute to the spreading of a cascade can be inferred using asynchronous updating. In this case, the propagation of influence is not instantaneous, but subject to a finite transit time. In the present study, transit times were assumed to be proportional to the ratio of projection length and projection weight. This operationalizes the idea that transmission along short, strong projections should be faster compared with long, weak projections. As a result, activations and signal spreading take place at nonuniform time intervals, and the model is updated asynchronously. Differences in transit times can then be used to infer, for any given node, exactly which of its incoming projections were used to propagate the cascade.

### Two Seeds, Same Signal

We next investigate scenarios where identical perturbations are initiated in two seed nodes simultaneously. If the resultant cascade spread is accelerated relative to the single-seed cases, this would indicate a synergistic relationship between the two seed nodes, that is, that those two nodes would potentially benefit from simultaneous stimulation due to the topology of the network. Specifically, we assess the spread time speedup ($S_{ij\to k}$) of a particular two-seed combination ($A_{ij\to k}$) relative to the faster of the individual single-seed scenarios ($A_{i\to k}$, $A_{j\to k}$):

$$S_{ij\to k}=\frac{\text{min}(A_{i\to k},A_{j\to k})-A_{ij\to k}}{\text{min}(A_{i\to k},A_{j\to k})}.$$ (6)

### Two Seeds, Different Signals

To investigate the evolution of multiple cascades, we extend the model to include two active states, for example, $r_{i}(t)=\left\{0,1,2\right\}$. Once a node adopts a particular state, it remains in that state indefinitely. For a node to adopt a particular state, its weighted inputs for that specific state must be greater than the threshold. Thus, inputs from competing states cannot be combined to exceed the threshold. If multiple competing states exceed the threshold for a particular node, the node adopts the state associated with the greatest total weight of its inputs.

Importantly, as the cascades develop, they eventually meet and form one or more fronts. In this scenario, we focus on the propensity of a node to mediate the integration of two cascades. To do so, we estimate the diversity of a node’s neighborhood as the entropy of its neighborhood vector. Specifically, for a set of *c* possible active states, the probability that the neighbors of node *i* will adopt state *x*_*c*_ is given by the entropy of its neighborhood:

$$h_{i}=-\underset{c}{\sum }P\left\{x_{c}\right\}\text{log}P\left\{x_{c}\right\}.$$ (7)

### Multiple Seeds, Multiple Signals

To investigate the propensity of nodes to coactivate, we extended the competitive spreading scenario described above to include more than two seeds. Specifically, we initiated two to *p* perturbations, each carrying a different signal. For any two nodes *i* and *j*, coactivation is defined as the propensity of that node pair to adopt the same signal. More formally, coactivation is defined as the number of times that nodes *i* and *j* adopted the same signal across all possible seeding scenarios. We performed this procedure for a range of scenarios, from *p* = 2 to *p* = 10 seeds. Since the number of possible seed combinations increases exponentially with the number of seeds, we sampled, without replacement, the space of all possible seed combinations by randomly choosing *m* = 10,000 pairs for each *p*-seed scenario.

## AUTHOR CONTRIBUTIONS

Jacob C. Worrell: Formal analysis; Visualization; Writing original draft. Jeffrey Rumschlag: Formal analysis. Richard F. Betzel: Methodology; Writing review & editing. Olaf Sporns: Conceptualization; Writing original draft; Writing review & editing. Bratislav Mišić: Conceptualization; Formal analysis; Methodology; Supervision; Visualization; Writing original draft; Writing review & editing.

## FUNDING INFORMATION

BM acknowledges support from the Natural Sciences and Engineering Research Council of Canada (NSERC Discovery Grant RGPIN #017-04265) and from the Fonds de recherche du Québec - Santé (Chercheur Boursier). OS acknowledges support from the J.S. McDonnell Foundation (#220020387), the National Science Foundation (#1212778), and the National Institutes of Health (NIH R01 AT009036-01).

## TECHNICAL TERMS

LPU (local processing unit): Neuronal population with common morphological and functional characteristics.
Linear threshold model: A dynamic model in which perturbations develop into signaling cascades and spread through a network of linked elements.
Sensors: Neuronal populations involved\break in transducing or processing\break sensory information.
Effectors: Neuronal populations involved\break in motor execution.
Spread time: Time required for a cascade initiated in a given source node to reach a target node.
Speedup: Decrease in spread time (acceleration) to a target node if a cascade is initiated in two source nodes as opposed to just one; quantifies potential for synergy between two nodes.
Diversity: The entropy of a node's neighborhood vector; quantifies the tendency for multiple cascades to converge at a particular node.
Coactivation: Probability for two nodes to be activated by the same cascade; quantifies the tendency for\break two nodes to engage in similar or overlapping functions.
